# Prevalence and characterization of *Salmonella* among humans in Ghana

**DOI:** 10.1186/s41182-017-0043-z

**Published:** 2017-02-10

**Authors:** Linda Aurelia Andoh, Shabana Ahmed, John Elmerdahl Olsen, Kwasi Obiri-Danso, Mercy Jemima Newman, Japheth Awuletey Opintan, Lisa Barco, Anders Dalsgaard

**Affiliations:** 1 Department of Veterinary Disease Biology, Faculty of Health and Medical Sciences, University of Copenhagen, Stigboejlen 4, 1870 Frederiksberg C, Denmark; 2 Department of Theoretical and Applied Biology, Kwame Nkrumah University of Science and Technology, Kumasi, Ghana; 3 Department of Microbiology, University of Ghana Medical School, Korle-Bu, Accra, Ghana; 4 OIE, National Reference Laboratory for Salmonellosis, Istituto Zooprofilattico Sperimentale delle Venezie, Legnaro Padova, Italy

**Keywords:** *Salmonella*, Antibiotic resistance, Serotypes, Phage types, Ghana

## Abstract

**Background:**

Non-typhoidal Salmonella (NTS) is a public health problem worldwide and particularly in Africa with high disease burden. This study characterized *Salmonella* isolates from humans in Ghana to determine serovar distribution, phage types, and antimicrobial resistance. Further, the clonal relatedness among isolates was determined.

**Methods:**

One hundred and thirty-seven *Salmonella* isolates (111 clinical and 26 public toilet) were characterized using standard serotyping, phage typing, and antimicrobial susceptibility testing methods. The molecular epidemiology of common serovars (*Salmonella* Typhimurium and *Salmonella* Enteritidis) was established by pulsed field gel electrophoresis (PFGE).

**Results:**

Twenty-two serovars were identified with *S*. Enteritidis, *S*. Typhimurium, and *Salmonella* Derby as the most dominant. One hundred and twelve isolates showed resistance to more than one antimicrobial. Fifty-eight (*n* = 58/112; 54.5%) strains were multi-resistant with low resistance to cephalosporins ceftazidime (8.0%), cefotaxime (4.5%), and cefoxitin (2.7%) with synergy to clavulanic acid indicating possible ESBLs. Isolates showed high resistance to trimethoprim (66.1%), tetracycline (61.6%), ampicillin (57.1%), sulfamethoxazole (46.4%), chloramphenicol (33.9%), and ciprofloxacin (25.0%). The most common resistance pattern of multi-resistant serovars was to ampicillin, chloramphenicol, sulphonamide, and trimethoprim. *S*. Enteritidis (18/43) strains reacted with typing phages but did not conform to any phage type with PT14B and PT4 as predominant definitive phage types. Six *S*. Typhimurium strains reacted but did not conform to any recognized phage type while seven were non-typable. The predominant definitive phage types were DT1 and DT22. PFGE patterns of human *S*. Enteritidis were closely related to patterns of poultry isolates obtained in a previous study in Ghana.

**Conclusions:**

Cephalosporin resistance is uncommon among *Salmonella* from humans in Ghana. Poultry may be an important source of human salmonellosis. There is an urgent need for the implementation of routine surveillance of antimicrobial use and bacterial resistance among humans in Ghana.

## Background

Non-typhoidal *Salmonella* (NTS) causes a high disease burden and a considerable number of deaths globally [1]. They are a leading cause of bacterial food-borne disease outbreaks and human gastroenteritis in developed countries and also a worry to public health in developing countries [1–3]. These medically important Gram-negative bacteria are a diverse group that can infect a wide range of animals and by transmission from this source can cause disease in humans [4]. In developed counties, NTS have been reported to mostly cause a self-limiting diarrheal illness in healthy individuals [5]. However, in sub-Saharan Africa, NTS is also a common pathogen isolated from the blood of both adults and children presenting with fever [1, 5–8]. NTS is known to have a fatality rate of 20–25% particularly in immune-compromised people [7]. Such cases are often mistaken as fever due to malaria; however, evidence now suggests that bacterial bloodstream infections accounts for most fevers of unknown etiology [9, 10]. Several investigations of outbreaks have implicated poultry and poultry products as a main source of human salmonellosis [11, 12]. Insufficient potable water supply and poor sanitation are also risk factors for food- and water-borne diseases associated with *Salmonella* serovars in developing countries.

In Ghana, 44.6% of poor households, particularly in urban areas, use public toilets with no hand washing facilities [13, 14]. The immediate surroundings of such toilets are also often polluted with both fecal and solid waste [13, 14]. On average, 1500 persons have been estimated to visit such toilets daily [13]. *Salmonella* has frequently been isolated from environmental sources that could serve as a link in its spread between different hosts [15]. The number of people sharing sanitation facilities among the poor creates unsanitary conditions and exposes the poor, children, and the immune-compromised to infectious diseases. In contrast to more developed countries, where food is mostly the main source of salmonellosis, direct human to human transmission is of more importance in less developed countries [16]. Pant and Mittal [17] reported 100% frequency of occurrence of *Salmonella* in fecal sludge at all stages of sewage treatment in Delhi, India. Similarly, the Ghana Health Service has indicated that about 80% of all out-patient department (OPD) cases such as cholera, typhoid, and diarrhea are sanitation and water related [18].

Over the years, reports around the world have shown *S*. Typhimurium and *S*. Enteritidis as the most common NTS serovars isolated from patients attending hospitals [19–22]. In Ghana, *Salmonella* Typhi has been reported as the predominant *Salmonella* serovar from bloodstream infections [6, 23]. The definitive phage type 1 (DT1) of *S*. Typhi has been identified in isolates from different geographical locations, indicating a possible clonal spread of this phage type within Ghana [6]. Infections due to NTS serovars are also a health problem in Ghana, with serovar Enteritidis and Typhimurium commonly isolated [6, 19, 24]. However, information on the sources, routes of transmission, serovar diversity in humans, and the zoonotic potential of *Salmonella* prevalence among food animals is still limited in Ghana, as in other developing countries, due to the lack of efficient epidemiological surveillance systems.

Non-typhoidal *Salmonella* infections mostly present with mild-to-moderate self-limiting gastroenteritis and antimicrobial treatment are only required in severe cases in immuno-compromised patients or invasive infections. However, due to increasing resistance of *Salmonella* to conventional antimicrobial agents, e.g., ampicillin and trimethoprim/sulfamethoxazole, used in the treatment of salmonellosis, amoxicillin/clavulanate, fluoroquinolones, and third-generation cephalosporins are now increasingly used. In Ghana, the Ghana Health Service recommends the use of chloramphenicol and ciprofloxacin as first-line drugs of choice for the treatment of invasive *Salmonella* infections, but in areas with multi-antimicrobial resistance (MAR), third-generation cephalosporins like cefotaxime or ceftriaxone as well as chloramphenicol or a fluoroquinolone are recommended. Lately, treatment with fluoroquinolones or third-generation cephalosporins has become common practice since ampicillin and cotrimoxazole have become ineffective due to resistance [6, 21, 23, 25].

In recent years, strains of *Salmonella* resistant to antimicrobial drugs have spread worldwide with isolates resistant to quinolones being reported with increasing frequency in several African countries [26–28]. In Ghana, earlier reports have indicated the presence of *Salmonella* strains showing multi-resistance to antimicrobials routinely used as therapeutic agents [24]. Of particular concern is the development of resistance to fluoroquinolones and extended-spectrum β-lactamses (ESBL) [29, 30] which may have been used either for prophylaxis, therapeutic treatment of humans, or as growth promoters in livestock feed. In Ghana, little is known about ESBL-producing *Salmonella* serovars.

The objective of the current study was to determine the serovar distribution of *Salmonella* from humans in Ghana, their phage types, and antimicrobial resistance. Further, the clonal relatedness of strains of the most common serovars was determined by pulse field gel electrophoresis (PFGE) and patterns compared to patterns of *Salmonella* isolates from poultry, which was the only food animal for which reliable *Salmonella* typing data were available in the country.

## Methods

### *Salmonella* strains collection

Presumptive positive *Salmonella* spp. isolates were collected from the central laboratory of the country’s largest referral hospital, Korle-Bu Teaching Hospital in the capital of Accra, and a hospital at the Kwame Nkrumah University of Science and Technology (KNUST) in Kumasi located northeast of Accra. The inclusion criteria was patients aged day 1 and above, patients with suspected septicemia or diarrhea, patients with bacteriologically confirmed *Salmonella* infection, or those presenting stool to the laboratory for examination.

Fecal sludge samples for *Salmonella* spp. analysis were collected from 12 public toilets located in three urban slum areas of Kumasi. These three urban settlements were selected because of their high poverty, poor and overcrowded housing, poor sanitation facilities, lack of access to quality health care, and inadequate management of large volumes of municipal solid waste [13]. Further, sampling fecal sludge from public toilets is also a cost-effective and convenient way of obtaining human *Salmonella* isolates. About 50 g of fecal sludge was obtained from the collection chamber of each toilet facility with a sterile plastic spoon and placed into labeled sterile plastic bags and transported to the laboratory in an ice chest with ice packs.

A total of 137 *Salmonella* isolates including 111 human clinical isolates and 26 isolates recovered from sludge collected from public toilets. The 111 human clinical isolates were recovered from blood (33) and stool (14) of patients seen at the Korle-Bu Teaching hospital in Accra and the University hospital in Kumasi between 2010 and 2011. The additional clinical isolates were 64 *Salmonella* isolates recovered from human blood/stool samples (specific source of origin unknown) between 2007 and 2009 at the Korle-Bu Teaching hospital.

### Isolation of *Salmonella* strains

#### Blood culture

Two milliliters of venous blood sample taken from patients was inoculated into 20 ml brain-heart infusion broth (BHI) (CM1135; Oxoid Ltd., England, UK). The culture bottles were incubated at 37 °C for 7 days and examined daily for evidence of bacterial growth, including turbidity and hemolysis. If bacterial growth was observed, a subculture was made after 24 h on blood agar (CM0271, Oxoid Ltd., England UK), Salmonella Shigella agar (SS) (CM0099, Oxoid Ltd., England, UK), and MacConkey agar (CM0007, Oxoid Ltd., England, UK).

#### Stool and fecal sludge culture

At the hospital, a swab of stool was inoculated into 45 ml buffered peptone water (CM0509 Oxoid Ltd., England, UK) and incubated at 37 °C for 18 h. In the laboratory, about 10 g fecal sludge sample from the public toilets was inoculated with a sterile plastic spoon into 90 ml buffered peptone water and incubated at 37 °C for 18 h. Then, 0.1 ml of the overnight culture was transferred into 10 ml of Selenite F broth that was homogenized and incubated at 37 °C for 18 h. Following incubation, the sample was streaked onto SS agar and SSI enteric media (Statens Serum Institute, Denmark) and incubated for 18–24 h at 35–37 °C. Transparent colonies with black centers on SS agar and cream colonies with metallic sheen and a black center due to H_2_S production on SSI enteric media were identified as presumptive *Salmonella*.

#### Biochemical identification, sero- and phage typing

All presumptive positive *Salmonella* isolates were subcultured onto nutrient agar plates (CM0309; Oxoid Ltd., England, UK) and incubated at 37 °C for 16–18 h. Isolates were first confirmed by their reaction in the Minibact-E biochemical tests (SSI, Denmark) and then by slide agglutination using polyvalent antisera (Poly A-E+Vi from SSI, Denmark). Specific serovars were established by serotyping at the WHO National *Salmonella* and *Shigella* Center, Institute of Health, Bangkok, Thailand [31].

Phage typing was done for *S*. Typhimurium (28 isolates) and *S*. Enteritidis (43 isolates) according to the scheme defined by the PHLS Colindale London at the OIE-National reference laboratory for *Salmonella*, Istituto Zooprofilattico Sperimentale delle Venezie, Italy [32]. Strains showing a typing pattern that did not conform to any recognized phage type was designated “reacted but did not conform” (RDNC) while those that did not show any phage type pattern at all were designated “non-typable” (NT).

#### Antimicrobial susceptibility testing

Antimicrobial susceptibility of *Salmonella* isolates was determined by the agar disc diffusion method on Mueller-Hinton agar (CM0337; Oxoid Ltd., England, UK) according to the protocol and guidelines of the European Committee on Antibiotic Susceptibility Testing (EUCAST). Test were carried out in Ghana and repeated in Denmark for quality assurance. The strains were screened for their susceptibility to the following antimicrobials: ampicillin (AMP, 10 μg), cefotaxime (CTX, 30 μg), cefoxitin (FOX, 10 μg), gentamicin (GEN, 10 μg), ceftazidime (CAZ, 30 μg), amoxicillin clavulanic acid (AMC, 30 + 10 μg), tetracycline (TET, 30 μg), chloramphenicol (CHL, 30 μg), trimethoprim (TRI, 5 μg), sulfamethazole (SUL, 240 μg), nalidixic acid (NAL, 30 μg), and ciprofloxacin (CIP, 5 μg) (ROSCO Diagnostic Neosensitabs, Denmark). *E. coli* ATCC 25922 and *Pseudomonas aeroginosa* ATCC 27853 were used for quality control. The EUCAST breakpoints [33] were used to interpret zone diameters.

Isolates that showed reduced susceptibility or resistance to ceftazidime were further investigated for ESBL phenotypes by a double disc diffusion test using the cephalosporins CAZ, CTX, and FOX alone and in combination with clavulanic acid. Interpretations were made according to the EUCAST breakpoints [33].

#### PFGE

PFGE genotyping was done for the dominant *Salmonella* serovars (52 strains of *S*. Enteritidis and 31 strains of *S*. Typhimurium) of human origin to establish genetic heterogeneity within serovar. PFGE patterns were compared with patterns previously described for *S*. Typhimurium (*N* = 1) serovar isolated from poultry [32]. Overnight culture of *Salmonella* spp. grown in Luria-Bertani (LB) broth (240230; Difco, MD, USA) was used to prepare genomic DNA according to the CDC PulseNet protocol [34] using 1% agarose (SeaKem ® gold agarose, Lonza, Rockland, ME, USA). DNA embedded in the agarose was digested with the restriction endonuclease XbaI (R0145; New England BioLabs, Inc). The DNA fragments were isolated by electrophoresis in 0.5× TBE buffer using CHEF DR III (Bio-Rad Laboratories, Hercules, CA, USA) system at 14 °C with initial switch time of 2.2 s, final switch time of 54.4 s, current 6 V/cm, included angle 120, and run time of 19 h. *Salmonella* Braenderup was used as the reference strain and a low range marker (NO350S; New England BioLabs, Inc.) used as the size marker. The gel was stained with 1% ethidium bromide solution for 30 min and de-stained in deionized water for 30 min. The gel image was captured by GelDoc EQ system with Quantity One® software (Version 4.2.1; Bio-Rad Laboratories, Hercules, CA, USA).

#### Data analysis

All data were entered into spreadsheet of Microsoft Excel 2010 and transferred to the statistical product for service solution (SPSS) (version16, 2008) for Windows which was used for descriptive analysis of data. Confidence intervals (95% CI) for prevalence of *Salmonella* strains found to be resistant to three or more antimicrobials were calculated as *ρ* ± z*square root of ((ρ*(1 − *ρ*)/*n*) where *ρ* is the estimated prevalence, *n* is the population size, and *z* = 1.96. Chi-square was used to test for differences in multi-resistant *Salmonella* proportion between blood and stool samples using Minitab software. Phylogenetic analysis of PFGE patterns was done using GelCompar® software (Version 4.6). The TIFF images were normalized by aligning the peaks of the size standard strain (*S*. Braenderup strain H9812) with the database global standard. Cluster analysis was performed by unweighted pair group method of the PFGE patterns using the Dice coefficient of 0.5% optimization with a 2.0% tolerance.

## Results

### *Salmonella* serovar diversity

All 137 strains were of *Salmonella enterica* and belonged to 22 serovars with *S*. Enteritidis (39.4%), *S*. Typhimurium (24.1%), and *S.* Derby (8.7%) as the most prevalent ones. *S*. 4, 5, 12: i: -, the monophasic variant of *S*. Typhimurium and *S*. Senftenberg was also observed (Table 1). *S*. Enteritidis and *S*. Typhimurium were more frequently isolated from human blood than stool samples from the hospitals, while *S*. Derby was the predominant serovar from fecal sludge.

**Table 1:** *Salmonella* Serovars isolated from human clinical and fecal sludge samples.

	Sample types				
Serovar	Stool^a^	Fecal sludge^b^	Blood^a^	Blood/stool^c^	No. of isolates (%)
*S.* Agona		1			1 (0.7)
*S.* Bredeney		1	1		2 (1.5)
*S.* Colindale	3		1		4 (2.9)
*S.* Derby		12			12 (8.8)
*S.* Dublin			3	2	5 (3.7)
*S.* Eastbourne			1		1 (0.7)
*S.* 4, 5, 12: i:-		1		1	2 (1.5)
*S*. 4, 12; -:1, 2	1				1 (0.7)
*S*. 9, 12: -:-				5	5 (3.7)
*S*. 8, 20: g, m:-	1				1 (0.7)
*S.* Enteritidis	1	4	9	40	54 (39.4)
*S.* Enugu	1				1 (0.7)
*S.* Ituri	1		1		2 (1.5)
*S.* Kaapstad		1			1 (0.7)
*S.* Oakland				1	1(0.7)
*S.* Oranienburg				1	1 (0.7)
*S.* Poona	2				2(1.5)
*S.* Senftenberg		1			1 (0.7)
*S.* Suberu		1		1	2 (1.5)
*S.* Typhimurium	2	3	18	10	33 (24.1)
*S.* Virchow	1		1	2	4 (2.9)
*S*. spp. (rough strain)		1			1 (0.7)
Total	13	26	35	63	137 (100)

^a^Blood and stool prospectively collected from patients at the two hospitals

^b^Fecal sludge collected from public toilets in slum communities

^c^Blood and stool retrospectively collected from patients at the Korle Bu teaching hospital

### Phage typing of *S*. Enteritidis and *S*. Typhimurium

Forty-six *S*. Enteritidis and 28 *S.* Typhimurium strains were phage typed. Forty-one percent (19/46) of *S*. Enteritidis strains reacted with phages but did not conform to any definitive phage type (RDNC). Seven recognized phage types were identified, with PT14B as the most common (10/46) (Table 2). This phage type and PT4 were common phage types shown by both hospital isolates and isolates obtained from fecal sludge samples. No common phage type was shared between the retrospective hospital strain collection and the *S*. Enteritidis isolated from the two hospitals during the current study.

**Table 2:** Phage type distribution of *Salmonella* Serovars Enteritidis and Typhimurium

	Sample types				
Phage type	Stool^a^	Fecal sludge^b^	Blood^a^	Blood/stool^c^	No. of isolates (%)
*S*. Enteritidis					
PT1			2	1	3 (6.5)
PT4		1	1	2	4 (8.7)
PT4B				1	1 (2.2)
PT8				1	1 (2.2)
PT14B		2		8	10 (21.7)
PT14C	1			3	4 (8.7)
PT34			1		1 (2.2)
RDNC			1	18	19 (41.3)
NT		1		2	3 (6.5)
Total	1	4	5	36	46
*S*. Typhimurium					
DT1				5	5 (17.9)
DT22	1		3		3 (10.7)
DT99			2		2 (7.1)
DT120				1	1 (3.6)
DT197		1		2	3 (10.7)
RDNC	1		4	1	6 (21.4)
NT		1	6		7 (25.0) 1
Total	2	2	15	9	28 2

^a^Blood and stool prospectively collected from patients at the two hospitals

^b^Fecal sludge collected from public toilets in slum communities

^c^Blood and stool retrospectively collected from patients at the Korle Bu teaching hospital

*S*. Typhimurium included six strains reacting but not conforming to any definitive phage type (RDNC), while seven strains were non-typable (NT). The predominant phage types were DT1, DT22, DT197, DT99, and DT120 (Table 2). DT197 was the only phage type common to both hospital and fecal sludge isolates of *S*. Typhimurium. DT22 was dominant among isolates collected from 2010 to 2011, while DT1 was the dominant phage type present among the retrospective isolates (Table 2).

### Antimicrobial susceptibility

Fifty-eight (42.3%) of the 137 *Salmonella* strains showed multiple antimicrobial resistance (MAR), i.e., they were resistant to three or more classes of antimicrobials, while one of the *Salmonella* from fecal sludge showed MAR. There was not one predominant resistance profile among hospital and fecal sludge strains except that the majority of the resistant strains shared common resistance pattern to AMP or TET (Table 3). Isolates showed low prevalence of cephalosporin resistance with 8.0% resistance to CAZ, 4.5% resistance to CTX, and 2.7% resistance to FOX. All of the ceftazidime- and cefotaxime-resistant isolates showed synergy with clavulanic acid indicating possible ESBL phenotypes. Isolates also showed high-level resistance to TMP (66.1%), SUL (46.4%), AMP (57.1%), TET (61.6%), CHL (33.9%), CIP (25.0%), and NAL (20.5%). MAR among isolates from blood (23/35) and stool (5/14) obtained from hospitals did not differ significantly (*χ* ^2^ = 3.675, *p* = 0.055).

**Table 3:** Antimicrobial resistance patterns of *Salmonella* serovars isolated from humans in Ghana

Serotype	*N*	Antimicrobials^a^												Summary^c^		
		TET	AMP	AMX	CIP	NAL	GEN	TMP	SUL	CHL	CAZ	FOX	CTX	0	1–3	>3
*S*. Enteritidis	54	32^b^	26	7	15	10	2	35	27	18	2	1	0	11	14	29
*S*. Typhimurium	33	11	16	3	5	3	1	17	12	11	2	2	2	4	10	19
*S*. Derby	12	11	11	0	1	0	0	0	0	0	0	0	0	1	11	0
*S*. Dublin	5	0	1	1	1	0	0	3	1	0	1	0	0	1	4	0
*S*. 9,12:-:-	5	4	3	0	1	1	0	4	4	3	0	0	0	1	1	3
*S*. Virchow	4	0	2	1	1	2	0	2	1	0	2	0	2	0	4	0
*S*. Colindale	4	1	1	1	0	1	0	2	0	0	1	0	0	2	1	1
*S*. Bredeney	2	1	0	0	0	0	0	1	1	1	0	0	0	1	0	1
*S*. 4,5,12:i:-	2	1	1	0	0	0	0	1	1	1	0	0	0	1	0	1
*S*. Poona	2	1	1	1	1	1	0	2	1	1	1	0	1	0	1	1
*S*. Ituri	2	1	0	0	0	1	0	1	1	1	0	0	0	0	1	1
*S*. Suberu	2	2	0	0	2	1	0	1	1	1	0	0	0	0	1	1
*S*. Agona	1	1	1	0	0	0	0	0	0	0	0	0	0	0	1	0
*S*. Eastborne	1	0	0	0	0	1	0	1	0	0	0	0	0	0	1	0
*S*. 4,12:-:1,2	1	0	0	0	0	0	0	0	0	0	0	0	0	1	0	0
*S*. 8,20:g,m:-	1	0	0	0	0	1	0	1	0	0	0	0	0	0	1	0
*S*. Enugu	1	0	0	0	0	1	0	1	1	0	0	0	0	0	1	0
*S*. Oakland	1	1	0	0	0	0	0	1	1	1	0	0	0	0	0	1
*S*. Oranienburg	1	0	0	0	0	0	0	0	0	0	0	0	0	1	0	0
*S*. Senftenberg	1	0	0	0	0	0	0	0	0	0	0	0	0	1	0	0
*S*. Kapstaad	1	1	0	0	1	0	0	1	0	0	0	0	0	0	1	0
*Salmonella* spp. (rough strains)	1	1	1	0	0	0	1	0	0	0	0	0	0	0	1	0
Total	137	69	64	14	28	23	4	74	52	38	9	3	5	25	54	58

^a^ *TET* tetracycline, *AMP* ampicillin, *CIP* ciprofloxacin, *NAL* nalidixic acid, *GEN* gentamicin, *TMP* trimethoprim, *SUL* sulfamethazole, *CHL* chloramphenicol, *AMX* amoxicillin clavulanic acid, *CAZ* ceftazidime, *CTX* cefotaxime, *FOX* cefoxitin

^b^Numbers under the different antimicrobials indicate the number of resistant isolates

^c^0 = susceptible to all tested antimicrobials, 1–3 = resistant up to three antimicrobials, >3 = multiresistant to more than three classes of antimicrobials

Among serovar *S*. Typhimurium, 19/33 (57.6%) were MAR with low prevalence of resistance to AMC, CAZ, CTX, and FOX and high resistance to TMP, SUL, CHL, AMP, and TET. The remaining 14 strains were either not resistant to any antimicrobial (4/33) or resistant to at most three antimicrobial (10/33) (Table 3). In general, *S*. Enteritidis strains showed the highest and most diverse resistance including 29/54 that were MAR. The most common resistance patterns of this serovar were AMP, TET, CHL, SUL, and TMP (6/29) and AMP, TET, SUL, and TMP (5/29) (results not shown). Eleven out of the 12 *S*. Derby strains isolated from public toilets showed resistance to AMP and TET (Table 3).

### PFGE genotyping

**Fig. 1:**
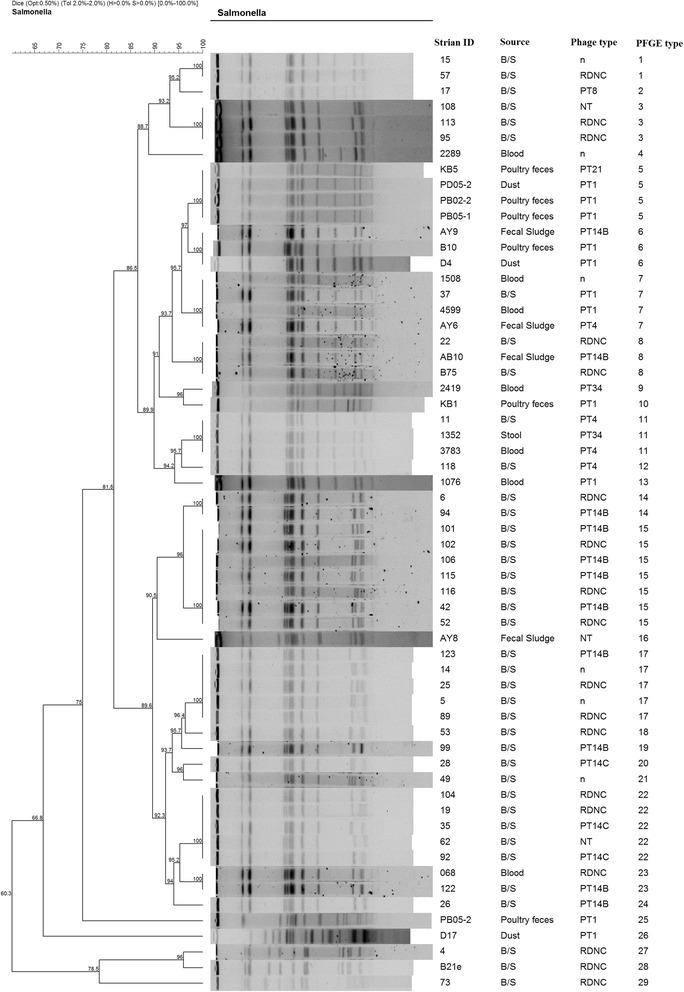
Dendrogram of cluster analysis of *S*. Enteritidis strains from human (B/S, blood, stool, and fecal sludge) and poultry (feces and dust) generated with gel Compar Software using unweighted pair-group arithmetic means (UPGMA) methods with 2.0% band position tolerance and 0.50% optimization parameter. *n* = not determined

**Fig. 2:**
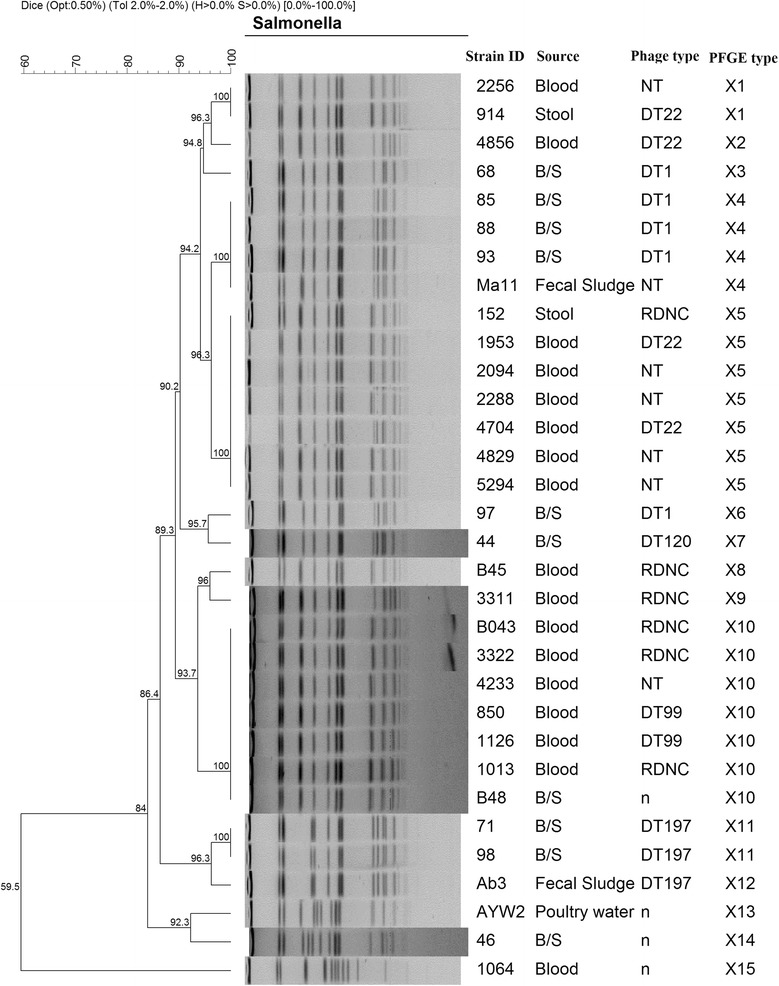
Dendrogram of cluster analysis of *S*. Typhimurium strains from human (B/S, blood, stool, and fecal sludge) and poultry generated with gel Compar Software using unweighted pair-group arithmetic means (UPGMA) methods with 2.0% band position tolerance and 0.50% optimization parameter. *n* = not determined

PFGE typing of XbaI-digested chromosomal DNA of the 83 strains belonging to *S*. Typhimurium (*n* = 31) and *S*. Enteritidis (*n* = 52) demonstrated that strains belonging to the same serovar were typically either identical or closely related with common band patterns irrespective of the source of isolation. Cluster analysis of *S*. Enteritidis strains showed wide genetic diversity with 25 PFGE types based on a one-band difference. Among these PFGE types, nine were represented by at least two strains clustering with 100% similarity index, the rest of the profiles were distinct (figure not shown). Cluster analysis of PFGE patterns of *S*. Enteritidis strains from humans and those from poultry (eight isolates) from a previous study [32] showed the strains were closely related genetically with two main clusters of 81.5% similarity (Fig. 1). Cluster I consisted of six retrospective strains and one strain from blood. Cluster II consisted of 20 strains of which 87.5% (7/8) of the poultry strains clustered together at 100% similarity index with strains from human sources while other strains from blood, stool, and fecal sludge clustered together or remained distinct with unique PFGE types. *S*. Typhimurium showed 15 different PFGE types with 5 represented by at least 2 strains (X1, X4, X5, X10, and X11) (Fig. 2). *S*. Typhimurium was not reported to be a common serovar among poultry isolates [32], and only one poultry *S*. Typhimurium strain was available for comparison. This strain showed a unique PFGE type but was 92.3% similar to a human strain (Fig. 2). Strains from blood and stool clustered together or remained unique irrespective of their source of isolation, phage types, or antimicrobial resistance. No correlation was observed between the phage types, antimicrobial resistance patterns, and the PFGE types of the isolates tested in this study.

## Discussion

Human salmonellosis is a major health problem in both developing and developed countries across the globe. NTS caused by salmonellae other than *S*. Typhi has been the leading cause of secondary bacteremia associated with gastroenteritis [8, 21]. We found 22 *Salmonella* serovars with *S*. Enteritidis and *S*. Typhimurium as the most dominant among hospital isolates while *S*. Derby was the most dominant serovar in fecal sludge. Generally, these serovars showed low resistance to cephalosporins and high resistance to other antimicrobials routinely used for therapeutic treatment of humans in Ghana.

In most parts of the world, surveys have reported *S*. Enteritidis and *S*. Typhimurium as the major serovars found in humans [35, 36]. In Ghana and most other African countries, they are also the most frequently isolated from bloodstream infections [7, 8, 21, 37] and from diarrheal diseases [38], which is confirmed by the findings of the present study. We report a low (8.0% CAZ, 4.5% CTX, and 2.7% FOX) cephalosporin resistance but high resistance to other antimicrobials among *Salmonella* strains, which calls for implementation of national surveillance systems of antimicrobial resistance and implementation of prudent antimicrobial usage, including revision of current national guidelines for antimicrobial usage due to the risk of resistance.

High-level cephalosporin resistance has been reported in *Salmonella* isolates from humans in countries like Malaysia [20], United States of America (USA) [39, 40], Jamaica [41], and Canada [42]. In Africa, high resistance has been reported to cephalosporins in Morocco [43], but on the other hand, low resistance has been reported in Burkina Faso [38]. A previous study in Ghana has reported no cephalosporin resistance in poultry from poultry farms and at poultry slaughter areas [32], since cephalosporins are not yet reportedly used in poultry production.

Over the years, ampicillin, chloramphenicol, and cotrimoxazole (trimethoprim/sulfamethoxazole) were first-line antimicrobials for treatment of severe salmonellosis [1, 44], but in developing countries like Ghana, chloramphenicol, fluoroquinolone, or third-generation cephalosporins are now commonly used as ampicillin, and cotrimoxazole have become ineffective due to development of resistance [25, 45]. Our susceptibility testing indicates moderate resistance to chloramphenicol (33.9%) and fluoroquinolones (25.0%), which means that although they are still a useful choice for empirical treatment of blood stream infections with *Salmonella* in Ghana, care must be taken to maintain or minimize increase in resistance.

*S*. Typhimurium has often been associated with multiple antimicrobial resistances [46] partly due to the emergence of *S*. Typhimurium definitive phage type (DT) 104 worldwide. Strains of this phage type are resistant to ampicillin, chloramphenicol, streptomycin, sulfonamides, and tetracycline [47]. Although *S*. Typhimurium DT 104 was not identified in this study, other phage types identified also showed high resistance to ampicillin, chloramphenicol, trimethoprim, and sulfonamides. It is interesting to note that most of the *S*. Enteritidis and *S*. Typhimurium strains were either RDNC or non-typeable by the phage typing system used, which indicates that new or undocumented phage types are emerging in developing countries and that such types can cause infections in humans. In this respect, previous reports that phage types of *S*. Enteritidis [48] may change as a result of acquisition of R-plasmids may be important, implying that selection for multiple antimicrobial resistant strains may change phage type distribution. PT14B, PT4, and PT1 were the dominant recognized phage types of *S*. Enteritidis, but with a time-dependent distribution. This picture of succession in dominance by particular phage types are also seen in other geographical regions. Point estimates of phage type distribution often show dominance of one type of *S*. Enteritidis, PT 29 in Nigeria, PT4 in England, PT6 in Denmark, PT8 in Poland, PT1 in Russia, and PT8 and PT13a in USA [49], but over time, they spread to other regions possibly due to trade and travel and are no longer so dominant. *S*. Typhimurium DT1 was previously identified as the dominant phage type in humans in Kenya [50], and apparently, this type is still highly prevalent, indicating a stable source for possible spill over to other humans.

Feglo et al. [51] reported a 2.3% *Salmonella* carriage among 258 healthy food handlers in Kumasi. Many food handlers and consumers, but also other people, use the public toilets [13]. Thus, the collection and analysis of fecal sludge from public toilets is a cost-effective and seemingly convenient and sensitive method to isolate *Salmonella* from human carriers with unknown disease status.

It is well recognized that antimicrobial-resistant *Salmonella* in poultry and other food products translate into resistance of *Salmonella* in humans. In order to investigate the role of poultry as a source for human NTS in Ghana, PFGE molecular typing analysis was done. PFGE has a high discriminatory power and is therefore often used in characterizing isolates from different sources in epidemiological studies of disease outbreaks [27, 52]. The similar PFGE band patterns generated in this current study among *S*. Enteritidis and *S*. Typhimurium (although just one isolate) indicate that poultry could be a likely source of *Salmonella* in humans in Ghana. The current study lacks parallelism between sampling in poultry and in hospitals, while concurrent sampling was performed in poultry and in public toilets in the same geographic region. Based on the overall lack of similarity in strains obtained from poultry and toilets, it seems fair to conclude that poultry most likely is far from the only source linked to human infections in Ghana. The most common *Salmonella* serovar found in poultry was *Salmonella* Kentucky [32]; however, this serovar was not observed among human isolates in our study although it has been reported earlier in humans in neighboring Burkina Faso [38]. This corresponds well with reports from other countries, USA for example, where *S*. Kentucky is also common in poultry, but very rarely causes human disease [53]. This lack of agreement between *S*. Kentucky prevalence in poultry and disease incidence in humans has been attributed to the distribution of some phage-associated virulence genes and virulence plasmids in *S. enterica* serovars [54]. Poultry associated *S*. Kentucky persists in poultry due to its metabolic adaptation to the chicken cecum but shows low virulence to humans, a situation that would be similar to what has been documented for the serovar Tennessee in Denmark [55]. Since the chain from poultry to humans is very short in Ghana, often consisting of just a slaughter process at local markets, the most likely explanation is that the poultry strain of *S*. Kentucky in Ghana is possibly of low virulence. The identical PFGE band patterns among the isolates of serovar Typhimurium and Enteritidis from humans (hospital and fecal sludge) indicate one or more common sources of exposure, e.g., food and water, which is not yet identified. *Salmonella* may even be transmitted directly from humans to humans, given the poor sanitary conditions in urban and peri-urban areas of Ghana. Thus, proper surveillance of all food animals and their products as well as humans for *Salmonella* is highly needed together with assessment of epidemiological risk factors associated with human salmonellosis.

The unsystematic and extensive use of antimicrobials in animal and human medicine has increased the emergence and spread of multiple antimicrobial-resistant bacterial pathogens. There is therefore an urgent need to establish a surveillance of possible circulating ESBL-producing and fluoroquinolone-resistant *Salmonella* in Ghana to ensure cautious use of antimicrobials in both human and animals. The genetic relationship between NTS isolated from humans (blood, stool, and fecal sludge) and poultry and the high prevalence of resistance to routine antimicrobials including fluoroquinolones as well as the seemingly low cephalosporin resistance observed in this study suggest that future studies should look more into NTS as causes of blood infections in Ghana. Sources and ways of transmission of antimicrobial resistance and MAR among NTS should be continuously monitored, e.g., in national microbiological and epidemiological surveillance programs.

## Conclusions

Possibly due to the unsystematic and extensive use of antimicrobials in animal and human, the level of multiple antimicrobial resistance in NTS in Ghana is high. There is therefore an urgent need to establish a surveillance of resistance in *Salmonella* in Ghana to assist in recommendations on the use of antimicrobials in both human and animals. The genetic relationship between NTS isolated from humans (blood, stool, and fecal sludge) and poultry and their high prevalence of resistance to routine antimicrobials including fluoroquinolones suggest that resistant NTS could be an important emerging public health threat in Ghana, e.g., as causes of blood infections. Sources and ways of transmission of antimicrobial resistance and MAR among NTS should be continuously monitored, e.g., in national microbiological and epidemiological surveillance programs.
